# The Effect of Comorbid Major Depressive Disorder on Working Memory in Young Adults With ADHD and the Mediating Role of the DLPFC

**DOI:** 10.31083/AP49137

**Published:** 2025-12-16

**Authors:** Qing-Juan Lai, Shi-Yu Zhang, Xin-Yi Zhang, Ning-Ning Liu, Wen-Chen Wang, Hai-Mei Li, Yu-Feng Wang, Lu Liu, Qiu-Jin Qian

**Affiliations:** ^1^Peking University Sixth Hospital/Institute of Mental Health, NHC Key Laboratory of Mental Health, Peking University, 100191 Beijing, China; ^2^National Clinical Research Center for Mental Disorders, Peking University Sixth Hospital, 100191 Beijing, China

**Keywords:** attention-deficit/hyperactivity disorder, dorsolateral prefrontal cortex, major depressive disorder, working memory

## Abstract

**Background:**

Attention-Deficit/Hyperactivity Disorder (ADHD) and Major Depressive Disorder (MDD) both exhibit working memory (WM) impairments and frequently co-occur. However, the impact of comorbid MDD on WM in ADHD patients and the underlying mechanisms remain unclear.

**Methods:**

The study included 409 adults, comprising 125 ADHD patients comorbid with MDD (ADHD+MDD), 145 ADHD patients without MDD (ADHD-MDD), and 139 healthy controls. In addition, functional connectivities (FCs) with the region of interest—the dorsolateral prefrontal cortex (DLPFC)—were analyzed in a subsample to explore the potential underlying neural mechanism.

**Results:**

The WM scores of the ADHD+MDD group were higher than those of the ADHD-MDD group. In all ADHD patients, depression scores were positively correlated with the WM impairment scores and explained 3.6% of the variance in WM impairment. Mediation analysis detected a potential effect of ADHD diagnosis on WM impairment via depressive symptoms. WM-related FC was identified between the left DLPFC and the right supramarginal gyrus (FC_[DLPFC/L - SMG/R]_), which partially mediated the relationship between the co-morbid status of MDD and WM.

**Conclusions:**

MDD in adults with ADHD exacerbated WM impairment, which may be related to the FC alteration between the left DLPFC and the right supramarginal gyrus (SMG). This finding provides a scientific basis for a deeper understanding of the pathogenesis and brain biomarkers of ADHD+MDD patients.

## Main Points

• Major Depressive Disorder (MDD) comorbidity worsens working memory impairment in Attention-Deficit/Hyperactivity Disorder (ADHD) patients.

• Depressive symptoms positively correlate with working memory deficits in ADHD.

• ADHD diagnosis affects working memory potentially via mediation of depressive symptoms.

• Functional connectivities (FC) between dorsolateral prefrontal cortex (DLPFC) and supramarginal gyrus mediates the MDD comorbidity effect on working memory (WM).

• This study provides new insights into brain biomarkers for ADHD+MDD.

## 1. Introduction

Attention-deficit/hyperactivity disorder (ADHD) is characterized by developmentally inappropriate inattention, hyperactivity, and impulsivity [1]. The pooled prevalence of ADHD in adults is 3.10% [2]. However, the identification and management of ADHD in adults remains inadequate. Changes in core symptoms or masking by compensatory strategies, prominent mood dysregulation or executive function-related symptoms, and extensive co-morbidities make the diagnosis of adult ADHD difficult [3]. In addition, adults with ADHD usually experience lower medication efficacy and have poorer tolerance to these treatments [4]. Overall, adults with ADHD have a poor prognosis.

Adult ADHD is associated with increased risk for psychopathology and impairment. Major depression disorder (MDD) is one of the most common co-morbidities. The estimated prevalence of MDD in the ADHD group ranges from 8.6% to 55% in the general population and 15.4% to 39.7% in the clinical population [5]. When ADHD patients are co-morbid with MDD (ADHD+MDD), the symptoms of each are more severe, the functional impairment is worse, and the treatment is more challenging [6]. How does the co-morbid status of MDD affect adults with ADHD, and what is the potential underlying mechanism?

The etiological mechanisms of ADHD have been extensively studied in recent years, with executive functions theory being one of the highly recognized neuropsychological theories [7]. Executive functions are responsible for managing and regulating a variety of complex mental activities, including subfunctions such as working memory (WM), inhibitory control, and cognitive flexibility. WM is a limited-capacity memory system that provides temporary storage and manipulation of information for advanced cognitive tasks [8]. WM plays an important role in interpreting ADHD symptoms [9] and may be one of the most critical factors in the pathogenesis of ADHD [10]. Meta-analysis suggested that WM is one of the important neurocognitive functions in patients with ADHD, with a mean effect size of up to 0.54 for the group difference between ADHD and healthy controls (HC), and a greater between-group difference in adults than in adolescents [11]. Similarly, WM deficits are a common feature in MDD and are associated with poor functional outcomes [12, 13]. The severity of WM impairment has been associated with the severity of the symptoms of depression [14, 15, 16].

The above evidence suggests that impairment of WM is a relatively important and shared executive-function impairment in both ADHD and MDD. However, the previous study has focused on these disorders independently, with limited research on WM in ADHD+MDD patients [17]. The existing literature presents mixed findings: some studies have suggested that comorbidity exacerbates WM difficulties [18], whereas others report no significant additive effect in either adolescent or adult samples [19, 20, 21]. This inconsistency is particularly notable since most research has focused on pediatric samples, leaving the high prevalence of MDD comorbidity in adult ADHD relatively underexplored. These mixed results highlight the need for more in-depth investigation of WM functioning in the comorbid condition of ADHD+MDD in adult populations. Particularly, to explain these clinically observed differences in cognitive performance, we need to move beyond behavioral-level research and explore potential neurobiological mechanisms that may mediate these WM deficits.

From a neurobiological perspective, the function of the dorsolateral prefrontal cortex (DLPFC) appears central to understanding WM impairments in both conditions. This region supports advanced cognitive functions including sequential WM processes [22, 23, 24]. Neuroimaging studies have linked DLPFC abnormalities to WM deficits in ADHD [25, 26, 27] and MDD [28, 29]. Specifically, MDD patients show more functional connectivity (FC) between the DLPFC and the inferior parietal lobule during WM tasks than do HC subjects [29]. Taken together with the existing evidence, we expected that functional abnormalities in the DLPFC may help to explain the effects ADHD+MDD on WM at the neurobiological level.

Altogether, MDD comorbidity in adult ADHD is common and challenging. WM deficits appear in both disorders, potentially representing a shared neuropsychological mechanism. DLPFC abnormalities likely contribute to WM impairments in the comorbid condition. Based on the studies cited, the hypothesis of the present study was: (1) comorbid MDD exacerbates WM impairment in adults with ADHD; the WM impairment in ADHD patients is influenced by the severity of depressed mood. And (2) the greater FC of the DLPFC could explain the influence of MDD or depressive symptoms on WM impairment in adult patients with ADHD. Therefore, the purpose of this study was to (1) explore the effects of comorbid MDD or depressive symptoms on WM in ADHD patients using covariance analysis (ANCOVA), partial correlation analysis, hierarchical regression analysis, and mediation analysis; and (2) explore the neural basis of the effects of comorbid MDD on WM in ADHD patients using FC analysis with the DLPFC as Region of Interest (ROI).

## 2. Methods 

### 2.1 Participants

ADHD patients were recruited from the outpatient clinic ofPeking University Sixth Hospital, China. HCs were recruited in communities through advertisements. All subjects were recruited between November 2018 and July 2023. The inclusion criteria of the ADHD group were as follows: (a) met the diagnostic criteria for ADHD, or ADHD+MDD according to the American Diagnostic and Statistical Manual of Mental Disorders (DSM-IV; American Psychiatric Association, 1994); ADHD patients were confirmed by the Conner’s Adult ADHD Diagnostic Interview for DSM-IV (CAADID) [30]; (b) at least 18 years old; (c) ADHD medication-naïve or had stopped taking ADHD medications for more than 5 half-lives; (d) IQ ≥90. The patients who underwent resting-state fMRI (rs-fMRI) scanning were also required to be right handed. The exclusion criteria for all participants were: (a) a history of schizophrenia, bipolar disorder, substance use related disorders, etc.; (b) a history of severe external brain injuries or neurological diseases, or had a history of other serious somatic diseases; or (c) identified suicide ideation. Patients undergoing rs-fMRI were also be excluded from having contraindications to fMRI scanning, such as metal implants in the body, suffering from claustrophobia or other conditions that make fMRI scanning unsuitable.

The inclusion criteria of the HC group were: (1) age ≥18 yr; and (2) IQ ≥90. HCs should not meet criteria for a history or current diagnosis of neurological or mental illness or serious somatic disease. The exclusion criteria for HCs undergoing rs-fMRI scanning were the same as for the patient group.

The study included 409 adults, comprising 125 ADHD+MDD patients (ADHD+MDD), 145 ADHD patients without MDD (ADHD-MDD), and 139 healthy controls.

### 2.2 Assessment Tools

#### 2.2.1 Structured Clinical Interview for DSM-IV Axis I disorders (SCID-I)

The interview assessed common mental disorders according to DSM-IV diagnostic criteria: mood disorder, schizophrenia and other psychotic disorders, substance abuse, anxiety disorders, somatoform disorders, eating disorders, and adjustment disorders [31].

#### 2.2.2 Conner’s Adult ADHD Diagnostic Interview for DSM-IV (CAADID)

According to the DSM-IV diagnostic criteria, individuals were diagnosed with adult ADHD if they met 5 or more of the 9 symptoms of inattention and/or 5 or more of the hyperactivity-impulsivity symptoms. These symptoms had to have been present for at least 6 mo and caused significant impairment in social functioning [30].

#### 2.2.3 Wechsler Adult Intelligence Scale (WAIS)

The WAIS is an internationally accepted IQ scale. The test consists of 11 subtests, and the total IQ was calculated based on the test results. Higher scores indicate higher levels of intelligence [32].

#### 2.2.4 Adult ADHD Rating Scale (ADHD-RS)

The ADHD-RS is a self-rating scale that assesses ADHD symptoms using DSM-IV diagnostic criteria. It allows for the calculation of inattention (IA) score, hyperactivity-impulsivity (HI) score, and total scale score, with higher scores being associated with more severe symptoms [33]. It has good reliability, with Cronbach’s alpha values of 0.81–0.88 for factor scores and total scores in children [34], and 0.89 in adults [35]. The present study used the IA factor, which yields a Cronbach’s alpha value of 0.96.

#### 2.2.5 Self-rating Depression Scale (SDS)

The SDS is used to assess the subjective feelings of depressed patients in the previous week. Higher scores indicat more severe subjective depression [36]. The scale has good reliability and was suitable for use in clinical work such as health surveys and outpatient screening [37]. Cronbach’s alpha values in non-intellectually disabled populations, worldwide, range from 0.73 to 0.93 [38]. In the present study, Cronbach’s alpha was calculated to be 0.90.

#### 2.2.6 Behavior Rating Inventory of Executive Function-adult Version (BRIEF-A)

The BRIEF-A can be used to assess impaired executive function in adults [39]. The scale consists of two main indices and 9 factors: inhibit, shift, emotional control, self-monitor, initiate, WM, plan/organize, organization of materials, and task monitor. The scale has been shown to have good reliability and validity, with Cronbach’s alpha values for its various factors ranging from 0.65 to 0.88 [40]. The present study used its WM factor to assess the degree of WM impairment in the subjects, which yielded a Cronbach’s alpha value of 0.92.

### 2.3 Resting-state fMRI (rs-fMRI) Data Acquisition and Preprocessing

The rs-fMRI data were collected using a 3T MR system (Discovery MR750; General Electric, Boston, MA, USA) in the Neuroimaging Center in the participating hospital. During the 8-min scan, participants were instructed to lie still with their eyes closed and maintain wakefulness while clearing their minds of thoughts. The rs-fMRI data were acquired using a gradient-echo, single-shot, echo-planar imaging (GRE-SS-EPI) sequence (detailed parameters in the “MRI Data Acquisition” section of the **Supplementary Materials**).

The left and right DLPFCs were selected as ROIs using the human Brainnetome Atlas (http://atlas.brainnetome.org). The seed points had MNI coordinates of (–27, 43, 31) and (30, 37, 36), with a radius of 6 mm. FC analysis was conducted using the RESTplus toolbox 1.30 and SPM12 software (https://www.fil.ion.ucl.ac.uk/spm/) on the MATLAB R2022b platform (The MathWorks, Inc., Natick, MA, USA) (details in the “Data Preprocessing” section of **Supplementary Materials**). The mean time series of each ROI was extracted, then Pearson’s correlation coefficients were calculated between the mean time series of the ROI and the time series of each voxel in the whole brain. FC was calculated separately for each hemisphere. Ultimately, the correlation maps were converted to z-value maps using Fisher’s z transformation.

### 2.4 Statistical Analysis

#### 2.4.1 Behavioral Analyses

Data were analyzed using SPSS 26.0 software (IBM Corp., Armonk, NY, USAIBM, USA). Statistical analyses were conducted using SPSS 26.0. Continuous variables are presented as mean ± standard deviation (SD), with normality assessed via Q-Q plots and evaluation of skewness and kurtosis. Between-group comparisons were performed using analysis of covariance (ANCOVA) with F-statistics and *p*-values for continuous variables, and χ^2^ tests with χ^2^ statistics and *p*-values for categorical variables. All tests were two-tailed with a significance level of α = 0.05.

From the categorical dimension, ANCOVAs were performed to compare the WM performance among the ADHD+MDD, ADHD-MDD and HC groups, using the Bonferroni correction for multiple comparisons. From the quantitative dimension, partial correlation analyses were conducted to explore the relationship of depressive symptoms and WM in ADHD and HC separately. Sex, age, and IQ were set as covariates for the above analyses. Because of the close relationship between inattention symptoms and WM [41], we set IA as a further covariate to explore the influence of MDD on WM more strictly. Then, to assess the relative contribution of depressive symptoms to WM, hierarchical regression analyses were performed in ADHD patients and HCs. Then, mediation analyses using PROCESS version 4.1 ( https://www.processmacro.org/index.html) were conducted to test a potential ‘ADHD→SDS→WM’ relationship.

#### 2.4.2 Imaging Analyses

First, we performed multiple regression analyses on the WM-related functional connectivities (FCs) in ADHD patients and HCs separately. The multiple comparisons in this section were corrected using the GRF correction method (set *p* < 0.001 at the voxel level, *p* < 0.05 at the cluster level and bilateral test; with post hoc analyses using the Bonferroni correction). Second, we compared the differences in WM-related FC between the ADHD+MDD and ADHD-MDD groups, and then analyzed the correlation of WM-related FC with IA and SDS scores. Finally, mediation analysis tested a potential ‘comorbid MDD/SDS/IA→FC→WM’ relationship.

#### 2.4.3 Further Analyses With ADHD+MDD Split into ADHD+cMDD and ADHD+pMDD

We further divided the ADHD+MDD group into ADHD patients currently comorbid with MDD (ADHD+cMDD) and those only previously comorbid with MDD (ADHD+pMDD). In the behavioral phase, we compared the demographic and symptom differences among the ADHD+cMDD, ADHD+pMDD, ADHD-MDD and HC groups. In the imaging phase, we compared the differences in WM-related FC among the three groups of ADHD patients. All analyses were conducted using Bonferroni correction for multiple comparisons.

#### 2.4.4 Sensitivity Analyses to Evaluate the Potential Confounding Influences of Comorbid Disorders 

Sensitivity analyses were performed to exclude the potential confounding effects of comorbidities other than MDD on the present results using two approaches. One was to set other comorbidity statuses as additional covariates. The other was to exclude subjects with these other comorbidities.

## 3. Results 

### 3.1 Behavioral Analyses

Disease-related information for ADHD patients is shown in Table 1.

**Table 1.** **The characteristic differences among three groups**.

		ADHD+MDD^1^	ADHD-MDD^2^	HC^3^	F/χ^2^	*p*	Post hoc analyses
n (Males, %)		125 (50, 40.0)	145 (74, 51.0)	139 (55, 39.6)	4.83	0.089	-
Age (Mean ± SD)		26.08 ± 5.94	26.45 ± 5.57	25.36 ± 3.22	1.71	0.182	-
IQ (Mean ± SD)		121.68 ± 8.32	122.00 ± 9.19	124.81 ± 7.28	5.87	0.003	1, 2 < 3
Edu. (yrs) (Mean ± SD)		14.10 ± 4.01	13.97 ± 3.50	16.99 ± 3.10	25.79	<0.001	1, 2 < 3
ADHD subtypes (n, %)							
	ADHD-I	68 (54.4)	86 (59.3)	-	1.64	0.440	
	ADHD-HI	0 (0.0)	1 (0.7)	-			
	ADHD-C	57 (45.6)	58 (40.0)	-			
Medication history (n, %)		8 (6.4)	5 (3.4)	-	1.28	0.259	
	OROS-MPH	7 (5.6)	3 (2.1)				
	ATX	1 (0.8)	2 (1.4)				
ADHD symptoms (Mean ± SD)							
	Total	29.58 ± 7.75	29.81 ± 8.40	23.76 ± 4.38	29.70	<0.001	1, 2 > 3
	IA	19.10 ± 4.09	18.60 ± 4.18	12.35 ± 3.07	126.21	<0.001	1, 2 > 3
	HI	10.48 ± 5.44	11.21 ± 5.99	11.38 ± 2.07	1.19	0.306	-
SDS (Mean ± SD)		58.70 ± 11.95	49.39 ± 10.70	35.13 ± 7.43	175.59	<0.001	1 > 2 > 3
WM (Mean ± SD)		20.03 ± 2.57	19.26 ± 2.53	9.53 ± 1.97	805.52	<0.001	1 > 2 > 3

ADHD+MDD^1^, ADHD patients comorbid with MDD; ADHD-MDD^2^, ADHD patients without MDD; HC^3^, healthy controls; IQ, Intelligence Quotient; Edu. (yrs), Education (years); OROS-MPH, osmotic-release oral system methylphenidate; ATX, Atomoxetine; ADHD-RS, Adult ADHD Rating Scale; IA, ADHD-RS inattention factor; HA, ADHD-RS hyperactivity/impulsivity factor; SDS, Self-rating Depression Scale; HI, hyperactivity-impulsivity.

#### 3.1.1 The Characteristic Differences Among 3 Groups of Subjects

For ADHD core symptoms, the distribution of IA scores was different among the three groups. Post hoc analyses indicated higher IA in the ADHD group than in the HC group (both *p* < 0.001); whereas no difference was found between the ADHD+MDD and ADHD-MDD groups (*p* = 0.751).

For depressive symptoms, the SDS scores were highest in the ADHD+MDD group, then the ADHD-MDD group, and the lowest in the HC group (all *p* < 0.001).

For the WM factor, a similar pattern to SDS score was found, in that ADHD+MDD > ADHD-MDD > HC (ADHD+MDD *vs* ADHD-MDD: *p* = 0.021; ADHD+MDD *vs* HC: *p* < 0.001; ADHD-MDD *vs* HC: *p* < 0.001).

#### 3.1.2 Relationships Between SDS Score and WM

**Fig. 1.**
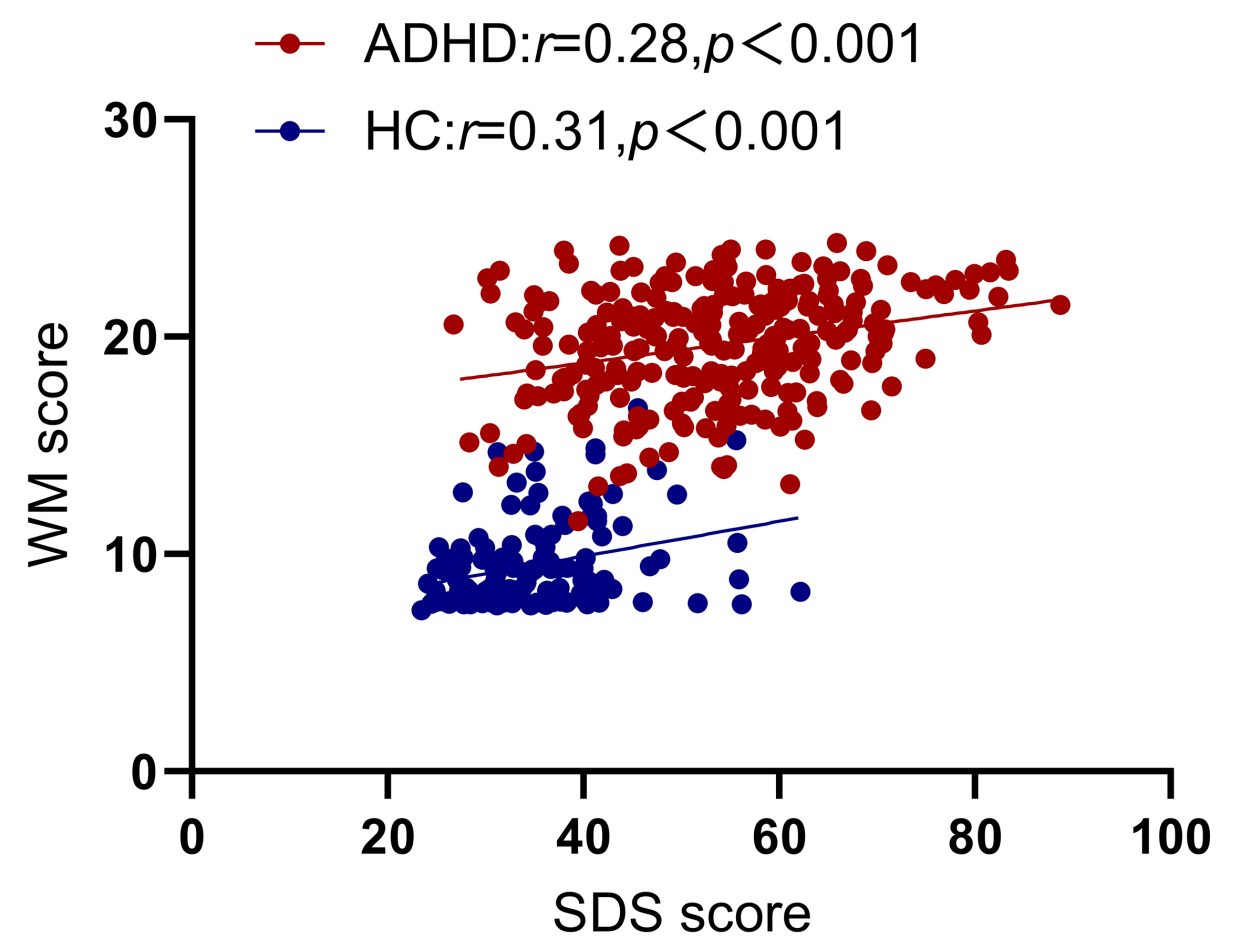
**Partial correlation analysis between working memory (WM) and Self-rating Depression Scale (SDS), with age, gender, and Intelligence Quotient (IQ) as covariates**. ADHD, Attention-Deficit/Hyperactivity Disorder; HC, healthy control.

**Fig. 2.**
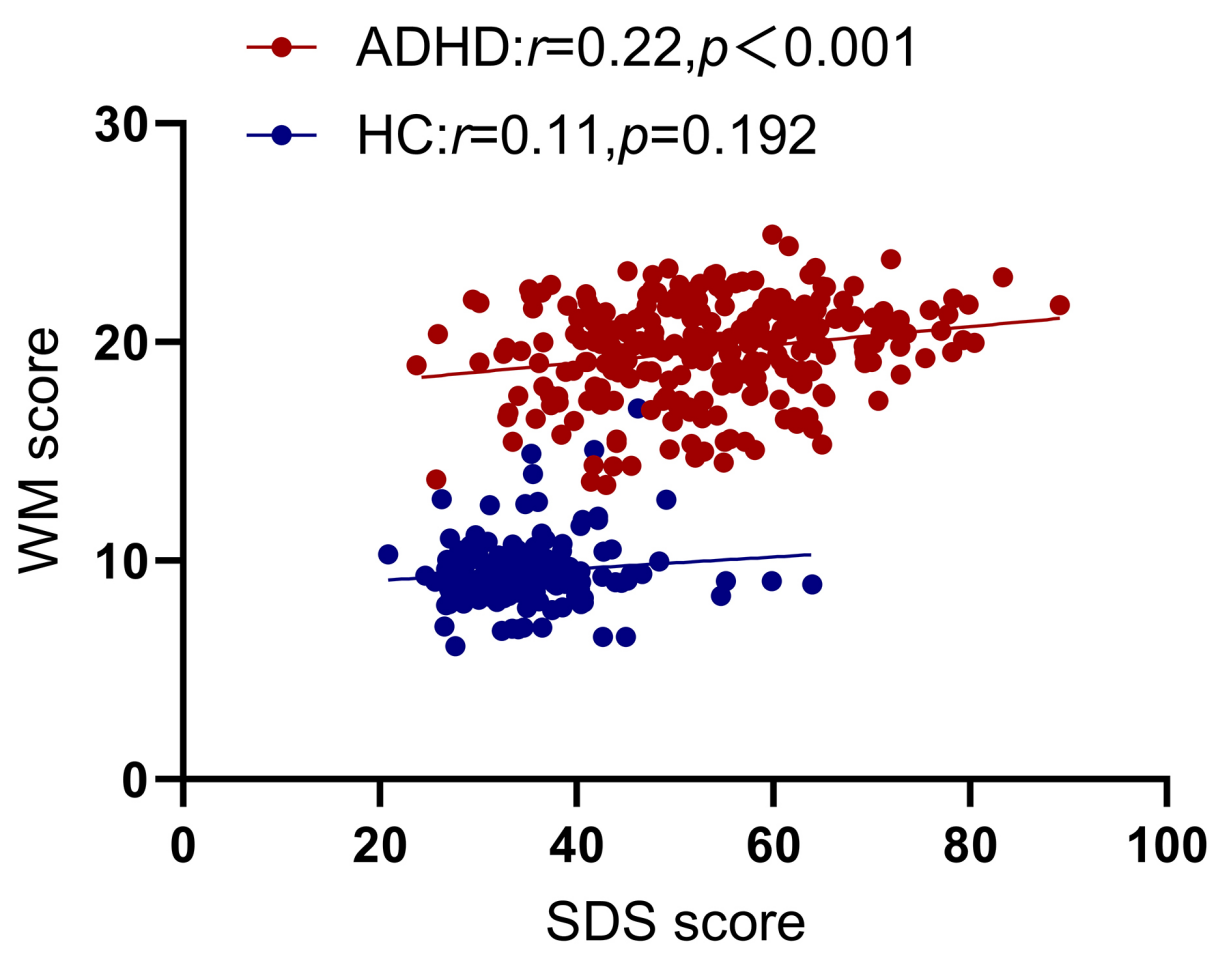
**Partial correlation analysis between WM and SDS, with age, gender, IQ and inattention (IA) as covariates**.

Significant correlation was found between WM and SDS scores both in ADHD patients (*r *= 0.28, *p* < 0.001) and HCs (*r *= 0.31, *p* < 0.001) (Fig. 1). However, when the IA score is also included as a covariant, the correlation between WM and SDS score was retained in ADHD patients (*r *= 0.22, *p* < 0.001), but not in HCs (*r *= 0.11, *p* = 0.192) (Fig. 2).

Hierarchical regression analyses in the ADHD group indicated that IA symptoms could explain 23.6% of the variance of WM (*p* < 0.001). With the existence of IA symptoms, the SDS score would explain 3.6% of the variance of WM independently (*p* < 0.001) (Table 2). It is interesting to note that in HCs, IA symptoms could explain 30.5% of the variance of WM (*p* < 0.001), whereas SDS scores could not explain an independent variance (*p* = 0.192) (Table 3). In both regression models, the variance inflation factor values for IA with control variables (IQ, age, sex), SDS score with control variables, and SDS score with IA plus control variables, were all below 1.2. That indicated that there was no significant multicollinearity among variables in either the ADHD or HC groups, confirming the reliability of the regression results.

**Table 2.** **The hierarchical Regression analysis of depressed mood and WM in ADHD group**.

Model	Variables	Coefficient	Standardized coefficient	*p*_1_	R^2^	F	*p*_2_	ΔR^2^	ΔF	*p*_3_
Model 1	Control Variables	22.96		<0.001	0.023	2.08	0.104	0.023	2.08	0.104
	gender	–0.57	–0.11	0.070						
	age	–0.04	–0.10	0.104						
	IQ	–0.01	–0.04	0.552						
Model 2	Control Variables	14.41		<0.001	0.259	23.13	<0.001	0.236	84.33	<0.001
	gender	–0.22	–0.04	0.427						
	age	–0.03	–0.06	0.295						
	IQ	0.00	0.01	0.831						
	IA	0.31	0.49	<0.001						
Model 3	Control Variables	12.32		<0.001	0.295	22.07	<0.001	0.036	13.47	<0.001
	gender	–0.41	–0.08	0.142						
	age	–0.02	–0.05	0.331						
	IQ	0.01	0.03	0.615						
	IA	0.28	0.46	<0.001						
	SDS	0.04	0.20	<0.001						

*p*_1_, Statistical tests corresponding to the model coefficients; *p*_2_, F Corresponding statistical tests; *p*_3_, ΔR^2^ Corresponding statistical tests.

**Table 3.** **The hierarchical Regression analysis of depressed mood and WM in HCs**.

Model	Variables	Coefficient	Standardized coefficient	*p*_1_	R^2^	F	*p*_2_	ΔR^2^	ΔF	*p*_3_
Model 1	Control Variables	9.96		0.006	0.023	1.04	0.376	0.023	1.04	0.376
	gender	0.56	0.14	0.104						
	age	–0.00	–0.00	0.991						
	IQ	–0.01	–0.04	0.654						
Model 2	Control Variables	4.90		0.111	0.328	16.34	<0.001	0.305	60.85	<0.001
	gender	0.32	0.08	0.274						
	age	0.03	0.04	0.569						
	IQ	–0.01	–0.03	0.703						
	IA	0.36	0.56	<0.001						
Model 3	Control Variables	4.09		0.190	0.336	13.49	<0.001	0.009	1.72	0.192
	gender	0.32	0.08	0.266						
	age	0.03	0.04	0.548						
	IQ	–0.01	–0.02	0.745						
	IA	0.33	0.52	<0.001						
	SDS	0.03	0.10	0.192						

*p*_1_, Statistical tests corresponding to the model coefficients; *p*_2_, F Corresponding statistical tests; *p*_3_, ΔR^2^ Corresponding statistical tests.

**Fig. 3.**
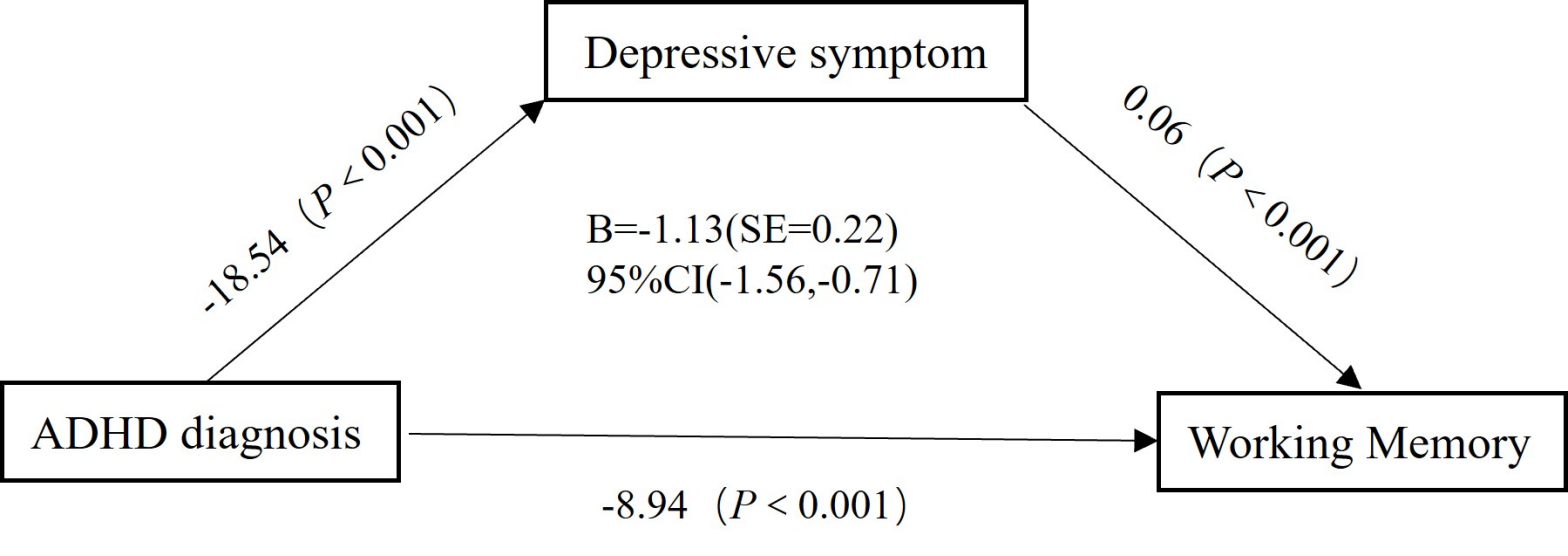
**Mediation analysis results with depressive symptom as the mediating variable, ADHD diagnosis as the independent variable, and working memory as the dependent variable**. SE, Standard Error.

Finally, we conducted a mediation analysis, which indicated a significant partial indirect effect of ADHD diagnosis on WM via SDS score [β = –1.13 (SE = 0.22), 95% CI = (–1.56, –0.71)] (Fig. 3). The proportion of the mediation effect was 0.112, indicating that approximately 11.2% of the effect was transmitted through depressive symptoms as a mediating variable. SDS score acted as a partial mediator between ADHD diagnosis and WM, even with IA symptoms controlled [β = –0.55 (Standard Error (SE) = 0.15), 95% CI = (–0.87, –0.27)].

### 3.2 Imaging Analyses

A total of 266 subjects completed rs-fMRI scans: 146 ADHD patients including 63 ADHD+MDD and 83 ADHD-MDD, and 120 HCs. Clinical and demographic information on this subset of subjects is shown in **Supplementary Table 1**.

**Fig. 4.**
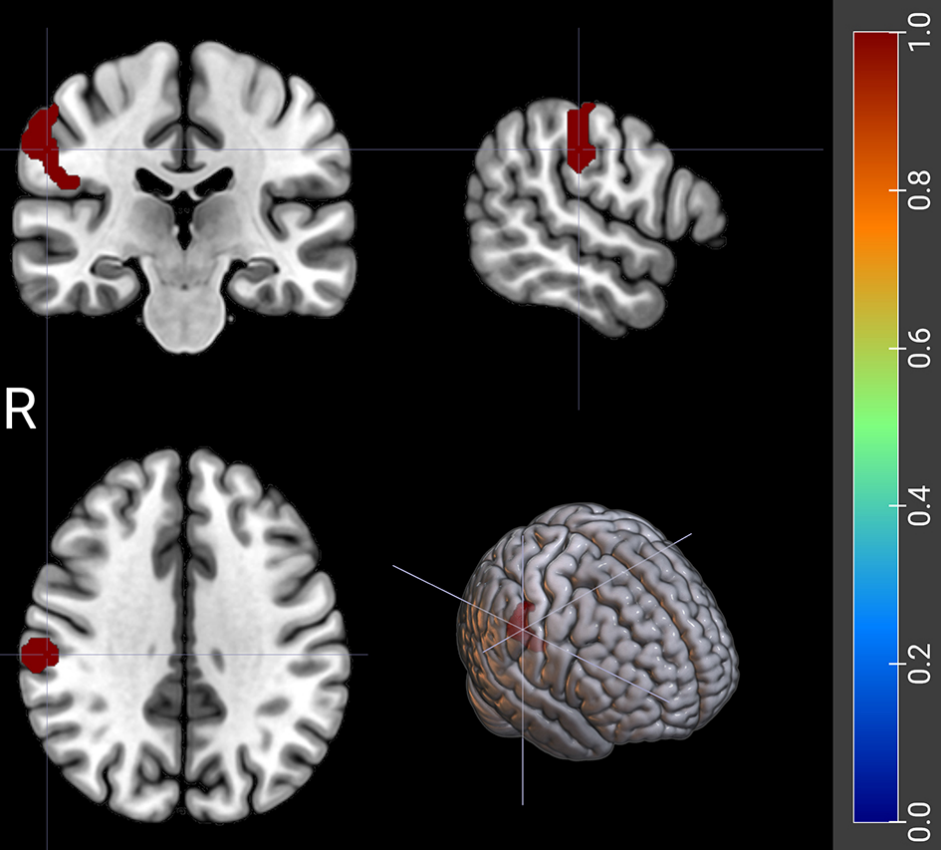
**The FC between left DLPFC and right supramarginal gyrus related to WM scores in ADHD patients**. The red shaded area represents the right supramarginal gyrus (SMG/R). DLPFC, dorsolateral prefrontal cortex; R, right side.

**Fig. 5.**
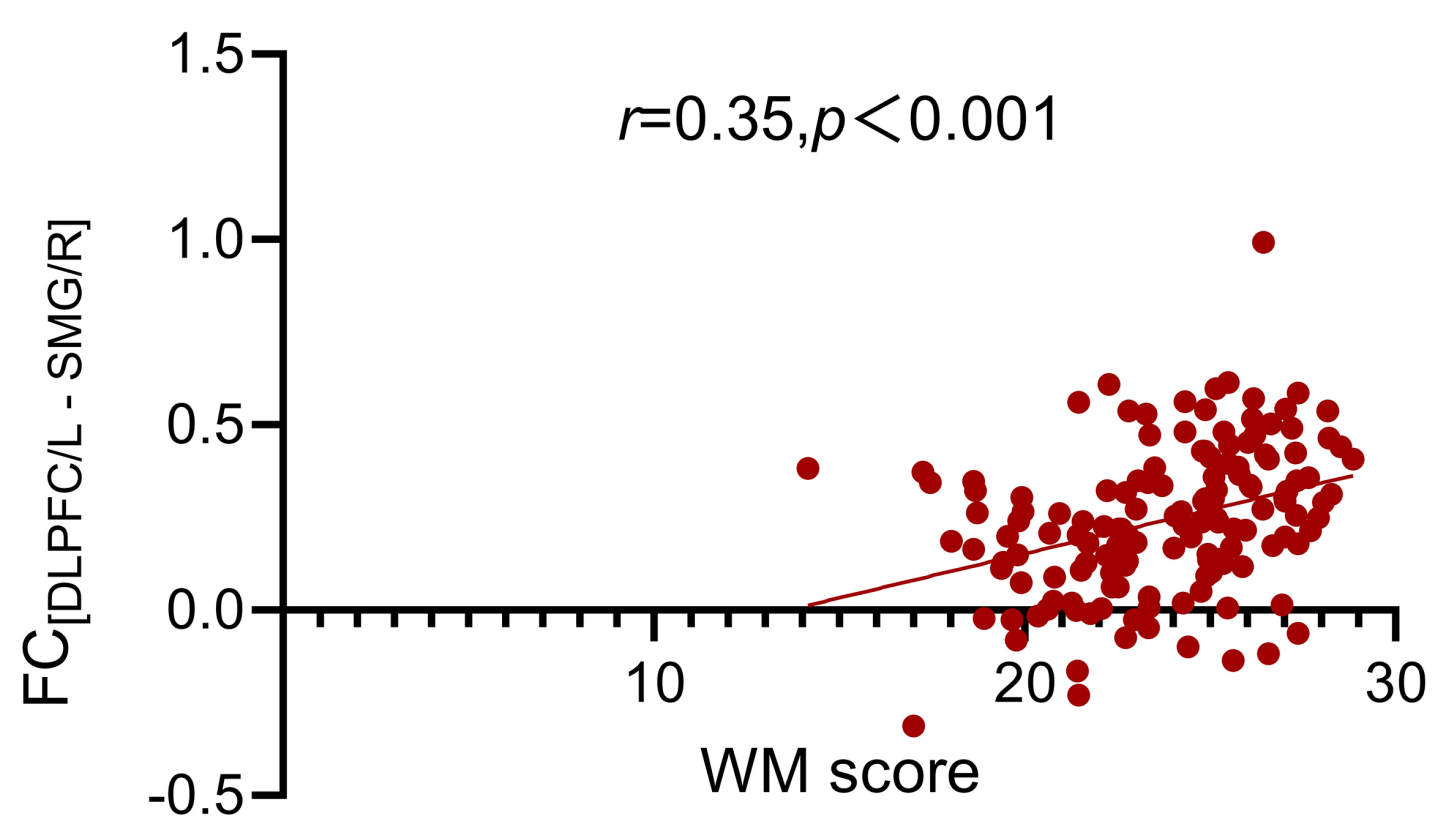
**The relationship between FC_[DLPFC/L - SMG/R]_ and WM scores in ADHD patients**. FC_[DLPFC/L - SMG/R]_, The FC between left dorsolateral prefrontal cortex and right supramarginal gyrus.

**Fig. 6.**
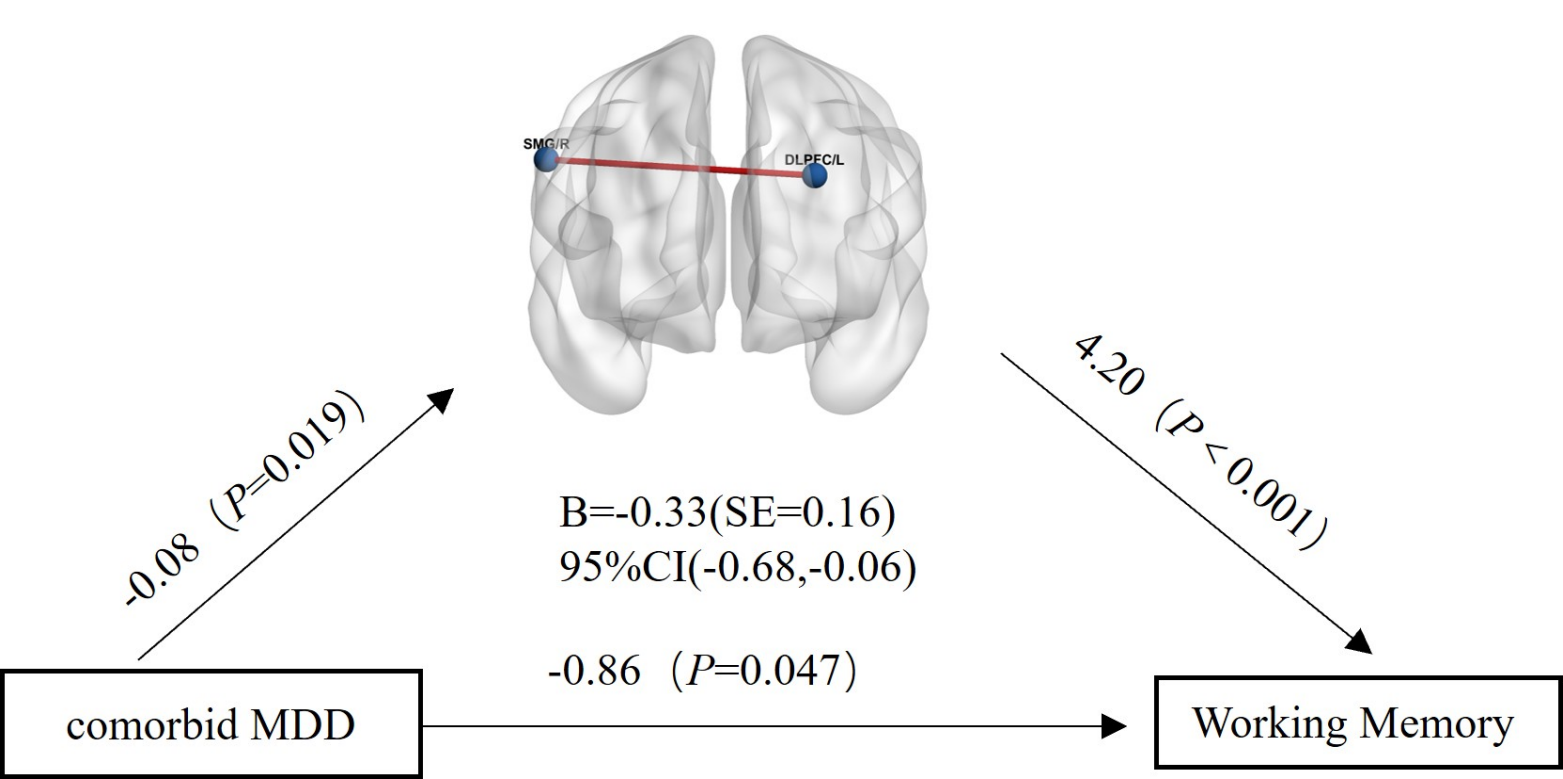
**Mediation analysis results with FC_[DLPFC/L - SMG/R]_ as the mediating variable, comorbid MDD as the independent variable, and Working Memory as the dependent variable**. SMG/R, right supramarginal gyrus.

In ADHD patients, WM-related FC was identified between the left DLPFC and the right supramarginal gyrus (SMG) (FC_[DLPFC/L - SMG/R]_, number of voxels: 149, Peak MNI coordinates: 57, –24, 36) (Figs. 4,5). It is interesting that the FC_[DLPFC/L - SMG/R]_ was found to be higher in the ADHD+MDD group than in the ADHD-MDD group (*p* = 0.019), and the result still existed after including IA as a covariate (*p* = 0.027). For symptom analyses, we did not find significant associations between the FC_[DLPFC/L - SMG/R]_ and with IA (*r *= 0.14, *p* = 0.089) (**Supplementary Fig. 1**) or SDS (*r *= 0.13, *p* = 0.134) (**Supplementary Fig. 2**). The mediation analysis showed that FC_[DLPFC/L - SMG/R]_ partially mediated the relationship between comorbid MDD and WM (95% CI = –0.68, –0.06) (Fig. 6).

**Fig. 7.**
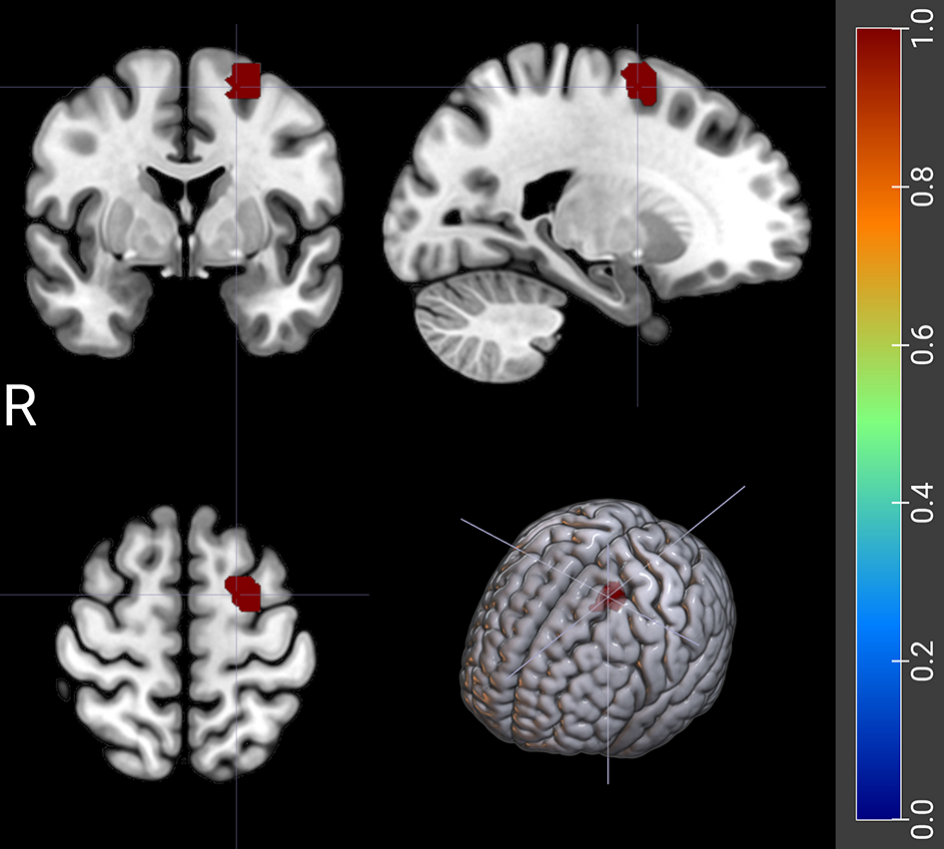
**The FC between left DLPFC and left superior frontal gyrus related to WM scores in HC**. The red shaded area represents the left superior frontal gyrus.

**Fig. 8.**
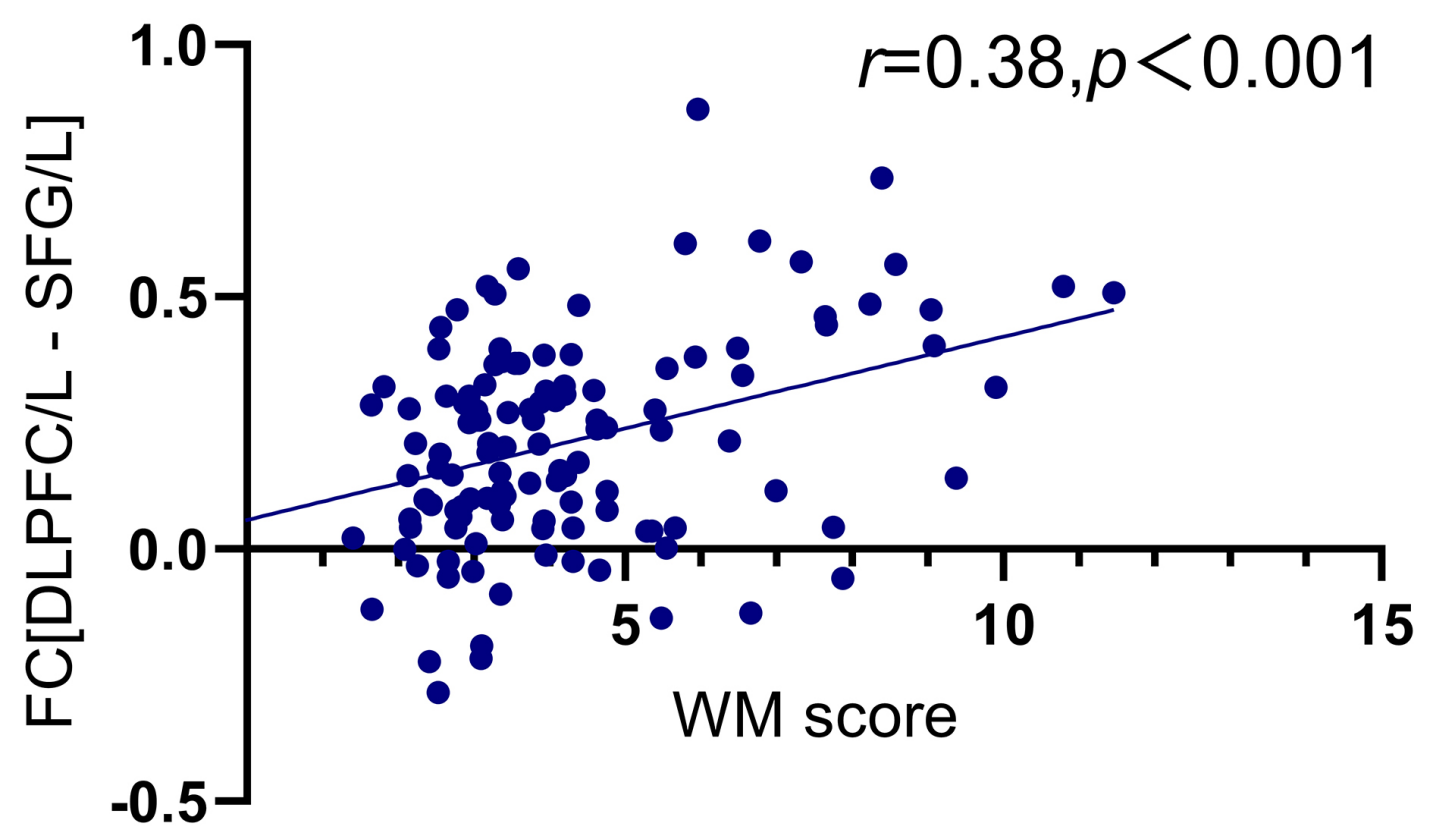
**The relationship between FC_[DLPFC/L - SFG/L]_ and WM scores in HC**. FC_[DLPFC/L - SFG/L]_, The FC between left dorsolateral prefrontal cortex and left superior frontal gyrus; WM, BRIEF-A working memory factor.

**Fig. 9.**
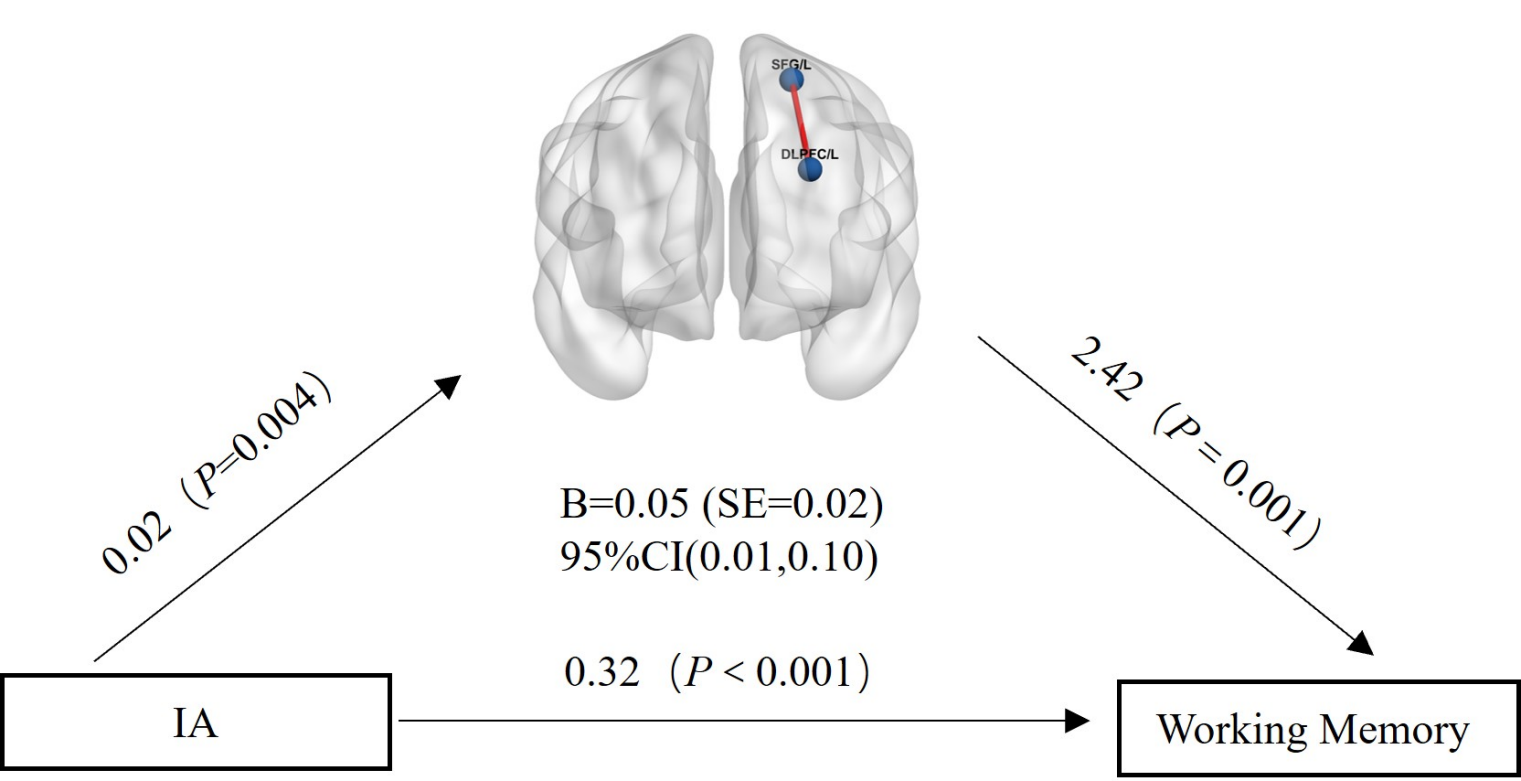
**Mediation analysis results with FC_[DLPFC/L - SFG/L]_ as the mediating variable, IA as the independent variable, and Working Memory as the dependent variable**.

In the HC group, WM-related FC was found between the left DLPFC and the left superior frontal gyrus (FC_[DLPFC/L - SFG/L]_, number of voxels:94, Peak MNI coordinates: –21, 0, 60) (Figs. 7,8). Similar to the behavioral results, this FC was significantly related to IA (*r *= 0.27, *p* = 0.004) (**Supplementary Fig. 3**) but not SDS score (*r *= 0.05, *p* = 0.594) (**Supplementary Fig. 4**). The FC_[DLPFC/L - SFG/L]_ could have partially mediated the relationship between IA and WM (95% CI = 0.01, 0.10) (Fig. 9).

### 3.3 Further Analyses With ADHD+MDD Split into ADHD+cMDD and ADHD+pMDD

A total of 49 individuals with ADHD+cMDD and 76 individuals with ADHD+pMDD completed the scale survey. Additionally, 23 individuals from the ADHD+cMDD group and 40 individuals from the ADHD+pMDD group completed the fMRI assessments.

#### 3.3.1 Behavioral Analyses

The mean IA score of ADHD patient groups was significantly higher than that of the HC group (all *p* < 0.001), but there was no significant difference among three groups within ADHD patients (all *p* = 1.000). SDS scores were highest in the ADHD+cMDD group, followed by the ADHD+pMDD group, then the ADHD-MDD group, with the HC group showing the lowest scores. Post-hoc analyses revealed significant differences between all groups (ADHD+pMDD *vs* ADHD-MDD: *p* = 0.008; all other pairwise comparisons: *p* < 0.001). WM scores of all three groups of ADHD patients were significantly higher than those of the HC group (all *p* < 0.001), with ADHD+cMDD scoring higher than ADHD-MDD within the ADHD patient groups (*p* = 0.011). However, there was no statistically significant difference between the ADHD+cMDD and ADHD+pMDD groups (*p* = 0.580), or between the ADHD+pMDD and ADHD-MDD groups (*p* = 0.829) (**Supplementary Table 2**).

#### 3.3.2 Imaging Analyses

No difference was found (*F *= 2.82, *p* = 0.063) in FC _[DLPFC/L - SMG/R]_ among the ADHD+cMDD, ADHD+pMDD and ADHD-MDD groups.

### 3.4 Sensitivity Analyses Considering the Other Comorbidities

The distribution of other comorbidities in ADHD is shown in **Supplementary Table 3**. When we removed the subjects with other psychiatric disorders or set the comorbid status of other psychiatric disorders as an additional covariate, the current findings were substantially retained (See **Supplementary Materials** for details).

## 4. Discussion

The present study was an attempt to elucidate the WM characteristics of ADHD+MDD patients, and explore its underlying neuroimaging mechanism. The findings present here suggest that the co-occurrence of MDD exacerbates WM impairment in ADHD patients, and depression severity affects WM performance. The WM-related FC between the left DLPFC and the right SMG partially mediates the relationship between the comorbid status of MDD and WM in ADHD patients.

The observed exacerbation of MDD or depressive symptoms on WM in adults with ADHD in the present study supported the findings reported previously [18, 42]. However, some controversy still exists, in that co-morbid MDD does not significantly impair WM in children or adolescents with ADHD [19, 20]. The inconsistent results may be due to the fact that our study was conducted with an adult sample. There are significant differences between childhood and adolescent MDD and adult MDD [43], and their effects on WM may also be different.

The subgroup analysis showed that current, rather than previous, comorbid MDD significantly aggravates WM impairment in adult ADHD patients. It confirms the existing evidence in the Mostert *et al*. [44] study that previous history of MDD does not exacerbate WM impairment in adults with ADHD. The possible reason is that on the one hand, it has been confirmed in the MDD population that there is no statistical difference in WM performance between the remission group and the HC group [45]. On the other hand, the study subjects were ADHD patients who already exhibited prominent WM impairment; the superimposed residual WM impairment during remission of MDD would obscure a significant difference. That suggests that WM impairment in patients with ADHD+MDD may be reversible, and that appropriate treatment as soon as possible is extremely necessary.

In terms of brain imaging, one of the brain regions most closely associated with WM is the DLPFC [23, 46], and this conclusion remains true when focusing on specific diseases such as ADHD [25, 26] and MDD [28, 29]. The present study showed that a functional alteration between the left DLPFC and the right SMG might play an important role in mediating WM impairment in adults with ADHD+MDD. This is the first time that the brain imaging mechanism of WM impairment has been directly explored in adult ADHD+MDD patients, which will help deepen the understanding of the comorbidity of the two and once again verify the role of the DLPFC in WM. The SMG is part of the inferior parietal lobule, and also part of the posterior parietal cortex, and is located in the 40th division of the Brodmann system. It has been reported to be associated with both spatial WM [47] and verbal WM [48]. SMG abnormalities have also been found to be associated with WM in MDD [49]. Our finding that FC abnormalities in the right SMG were associated with WM impairment in adult ADHD+MDD patients is consistent with previous conclusions.

Both the DLPFC and the SMG are important brain regions of the frontoparietal network (FPN) [50]. Active tasks involving WM and external thinking are an important function of the FPN [51]. Previous research has identified a mechanistic link between FPN global efficiency and working memory deficits in both ADHD [27] and MDD [12] populations. We found that adult ADHD+MDD patients could mediate WM impairment via enhanced FC between the right SMG and left DLPFC; this is consistent with previous studies. This enhanced FC may reflect a neural mechanism in which the brain attempts to compensate for the inefficiency of the basic WM network by recruiting additional neural resources. This pattern aligns with previous observations in ADHD adults who demonstrated FPN overactivation during low-intensity WM tasks, although this compensatory mechanism appears to fail when WM demands exceed capacity, resulting in impaired left FPN activation [27]. The present study revealed the role of the FPN in ADHD+MDD, providing a scientific basis for a deeper understanding of the pathogenesis and brain biomarkers of ADHD+MDD.

There is no doubt that inattention symptoms are closely correlated to WM impairment [41]. We have also verified that IA and WM scores are significantly positively correlated in both ADHD patients and HCs. Depressive symptoms have been traditionally associated with WM dysfunction in terms of information encoding and retrieval [52]. As with findings in the MDD group [14, 15], our results suggest that WM impairment in ADHD patients worsens with increasing levels of depression even after controlling for inattention symptoms. It is interesting to note that in the HC group, SDS score was significantly related to WM only when inattention symptoms were not controlled for. That indicates that although depressive symptoms can affect WM in HCs, the impact might not be as profound as in ADHD patients because HCs do not have the compounded effect of ADHD-related cognitive dysfunction. HCs generally have better baseline cognitive function, so the additional burden of depression, although significant, might not impair WM to the same extent as it does in ADHD patients. It is also worth pointing out that no significant effect of comorbid MDD on ADHD inattention symptoms was found in our study. We propose two possible explanations for this observation. First, MDD may have a more direct effect on WM, which is the underlying neuropsychological factor in ADHD patients [9]. This direct effect on WM may overshadow any potential influence of MDD on the inattention symptoms of ADHD. Second, ADHD patients themselves have severe inattention symptoms, and the additional aggravation of inattention caused by MDD might be obscured.

Another difference between ADHD subjects and HCs was the WM-related brain functional alteration. For HCs, the FC between the left DLPFC and the left SFG was associated with WM, and it could have partially mediated the relationship between IA score and WM. Although it was different from the finding in ADHD patients in our study, it was consistent with previous literature [53, 54]. The SFG has been implicated in WM, particularly in the maintenance of information without the need for manipulation [55]. This indicates that the WM-related brain areas in individuals with ADHD may differ from those in HCs. However, it is important to interpret these findings cautiously, as differences observed might also stem from variations in research samples.

This study has several advantages. First, it is the first study to explore the impact of current and previous comorbid MDD and depression severity on WM in an ADHD sample. Second, this study contains both behavioral data and brain imaging data, and deeply explores the brain imaging basis of WM in patients with ADHD comorbid with MDD. Third, the research object of this study is the adult ADHD group, whereas previous studies have mostly focused on children and adolescents [18, 19, 20]. Therefore, this study helps to promote the understanding of the WM performance and the rs-fMRI basis of comorbid MDD in the adult ADHD group.

However, our study also has some limitations. First, we did not include a separate MDD group, and all analyses were performed in patients with ADHD, which limits the interpretation of the results. Second, to measure WM, we relied solely on self-report scales rather than on objective tests. Self-report measures such as BRIEF and SDS are subject to inherent limitations including cognitive biases, social desirability effects, and limited insight of participants into their own cognitive processes. Although ecological cognitive function assessment may be more diagnostic than laboratory functional assessment [56, 57], the lack of objective performance-based measures limits our ability to fully capture WM functioning. The integration of both self-report and objective test results would have provided more comprehensive and methodologically robust findings. Third, subjects in this study, both patients and HCs, were younger and had higher IQs than would be expected in the general population. Perhaps young and highly intelligent people are more susceptible to WM impairment in their study and work and have better medical conditions. Therefore, future studies should consider recruiting the general population with a wider age range in the community. Fourth, we excluded participants on ADHD medications but not those using other psychiatric drugs, nor did we track such usage. These medications could affect cognition and neural activity, potentially confounding results. Future research should better control for or document medication effects to improve reliability and interpretability.

## 5. Conclusions

The present study demonstrated that comorbid MDD aggravates the WM impairment in ADHD patients, and the more severe the depressive symptoms, the more serious the WM impairment. The impaired WM in adult ADHD+MDD patients may be related to the abnormally enhanced functional connectivity between the left DLPFC and the right SMG. The present findings furthered the understanding of the effects and possible mechanisms of comorbid MDD on WM in adults with ADHD. From a clinical perspective, this study also suggests that the identification and treatment of depressed mood in patients with ADHD is crucial, and that timely interventions not only improve patients’ mood but also may contribute to improving their WM.

## Availability of Data and Materials

The data and materials that support the findings of this study are available from the corresponding author upon reasonable request.

## Supplementary Material

Supplementary material associated with this article can be found, in the online version, at https://doi.org/10.31083/AP49137.
