# Supplementary material for: The Effect of Comorbid Major Depressive Disorder on Working Memory in Young Adults With ADHD and the Mediating Role of the DLPFC
Source: Alpha Psychiatry. 2025 Dec 16;26(6):49137. doi: 10.31083/AP49137 (PMC12781219; doi:10.31083/AP49137)
Supplement: Supplementary file 1 [file 2757-8038-26-6-49137-s1.docx]

**Supplementary methods**

**MRI Data Acquisition**

The 8-min resting-state functional data were acquired using a gradient‐echo single‐shot echo-planar imaging (GRE‐SS‐EPI) sequence with the following parameters: repetition time (TR)= 2000 ms, effective echo time (TE)=30 ms, flip angle (FA) = 90°, slice thickness = 3.2 mm with no gap; matrix = 64 × 64; field of view (FOV) = 220 × 220 mm; 43 axial slices and 240 volumes. High‐resolution T1‐weighted anatomical images were acquired with the following parameters: 128 slices, slice thickness = 1.33 mm; TR= 6.66 ms; TE = 2.93 ms; TI = 450 ms; FA= 8°; FOV = 256 × 256 mm; matrix = 256 × 256; slice thickness = 1.0 mm with no gap, 180 sagittal slices.

**Data Preprocessing**

The preprocessing procedure for FC consisted of(1) exclusion of the first 10 time points to exclude the effects on the results caused by subject discomfort at the beginning of the scan and inhomogeneity of the magnetic field; (2) temporal layer correction to eliminate the discrepant images due to the different time of acquisition of data from different parts of the brain; (3) head movement correction to exclude subjects with head movements >3 mm or rotations >3°; (4) spatial normalization, first to the EP1 template, then functional images normalized to Montreal Neurological Institute (MNI) space (resampled voxel size = 3 × 3 × 3 mm3); (5) spatial smoothing, (6) linear detrending, (7) interference covariate regression: including 24 head movement-related parameters, white matter signal, and cerebrospinal fluid signal, and (8) filtering (0.01-0.08 Hz).

**Supplementary results**

**Supplementary Table 1. The characteristic differences among three groups completing fMRI scans.**

|  | **ADHD+MDD ^1^** | **ADHD-MDD ^2^** | **HC ^3^** | **F/χ^2^** | ***p*** | **Post hoc analyses** |
| --- | --- | --- | --- | --- | --- | --- |
| **n(Males, %)** | 63(26 ,41.3) | 83(39 ,47.0) | 120(47 ,39.2) | 1.26 | 0.534 | - |
| **Age(Mean ± SD)** | 25.75 ±5.48 | 26.40±5.76 | 25.41±3.22 | 1.09 | 0.338 | - |
| **IQ(Mean ± SD)** | 121.84±7.52 | 122.95±9.06 | 125.20±6.54 | 4.58 | 0.011 | 1 < 3 |
| **ADHD subtypes (n, %)** | | | | | | |
| ADHD-I | 37 (58.7) | 51 (61.4) | - | 0.11 | 0.740 |  |
| ADHD-HI | 0 (0.0) | 0 (0.0) | - |  |  |  |
| ADHD-C | 26 (41.3) | 32 (38.6) | - |  |  |  |
| **Medication history (n, %)** | 2(3.2) | 2(2.4) | - | 0.08 | 0.779 |  |
| OROS-MPH | 2(3.2) | 2(2.4) |  |  |  |  |
| ATX | 0(0.0) | 0(0.0) |  |  |  |  |
| **ADHD symptoms (Mean ± SD)** | | | | | | |
| total | 29.19 ± 8.48 | 29.36 ± 9.37 | 23.69± 4.26 | 18.81 | ＜0.001 | 1, 2 > 3 |
| IA | 19.08 ± 4.30 | 18.27 ± 4.40 | 12.28 ± 2.89 | 91.95 | ＜0.001 | 1, 2 > 3 |
| HI | 10.11 ± 5.90 | 11.10 ± 6.34 | 11.41 ± 2.04 | 1.73 | 0.179 | - |
| **SDS (Mean ± SD)** | 57.95±9.71 | 45.34±8.46 | 34.54±6.75 | 180.82 | ＜0.001 | 1 > 2 > 3 |
| **WM (Mean ± SD)** | 20.11± 2.68 | 18.88 ± 2.60 | 9.53 ± 1.98 | 573.81 | ＜0.001 | 1 > 2 > 3 |
| **FC _[DLPFC/L - SMG/R]_** | 0.26±0.23 | 0.19±0.18 | - | 4.34 | 0.019 | 1 > 2 |

ADHD+MDD^1^: ADHD patients comorbid with MDD; ADHD-MDD^2^: ADHD patients without MDD; HC^3^: healthy controls; IQ: Intelligence quotient; OROS-MPH: osmotic-release oral system methylphenidate; ATX: Atomoxetine; ADHD-RS: Adult ADHD Rating Scale; IA: ADHD-RS inattention factor; HA: ADHD-RS hyperactivity/impulsivity factor; SDS: Self-rating Depression Scale; WM: Behavior Rating Inventory of Executive Function-Adult Version working memory factor.


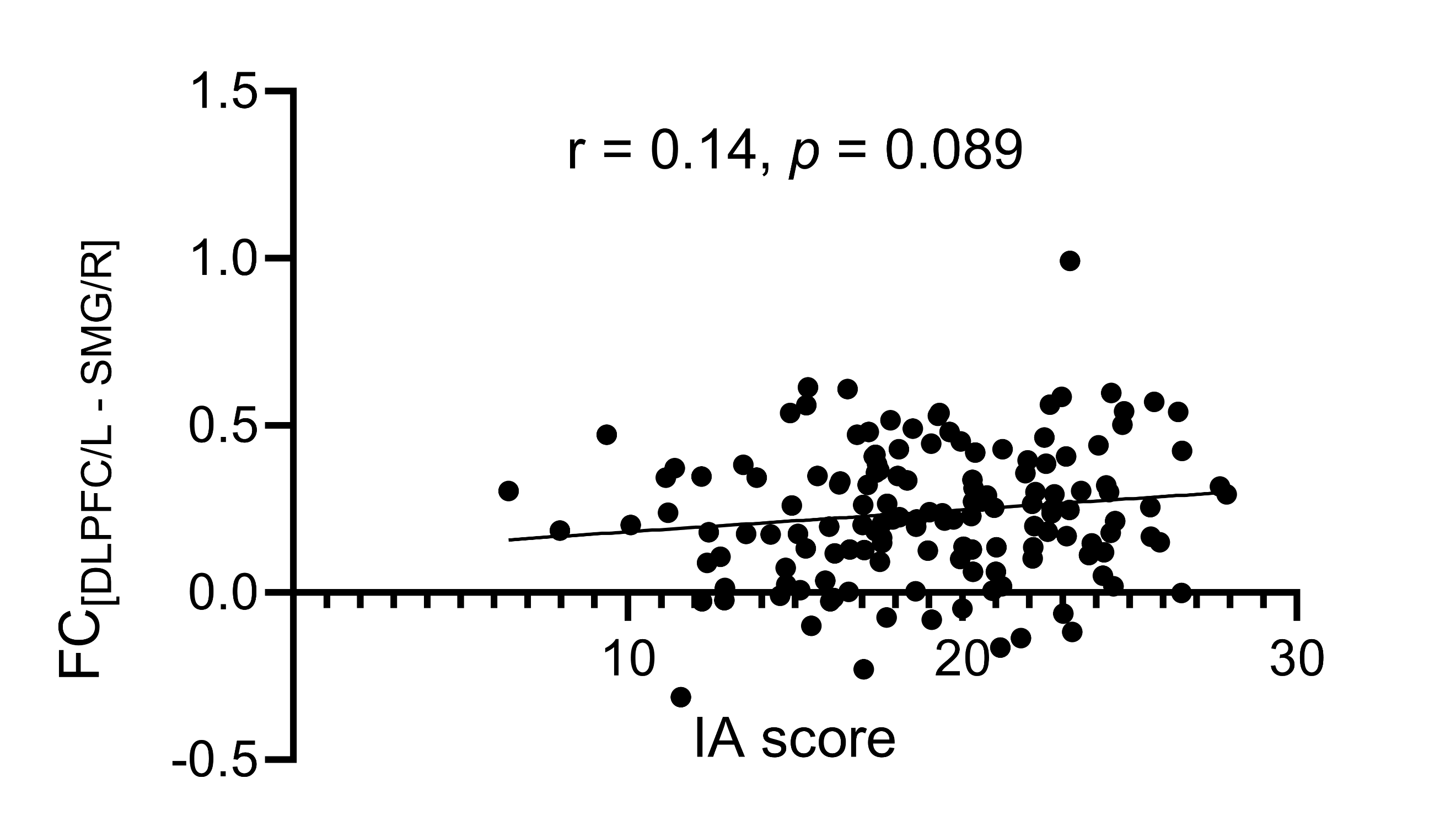


**Supplementary Fig. 1. The relationship between FC _[DLPFC/L - SMG/R]_ and IA scores in ADHD patients.**

FC _[DLPFC/L - SMG/R]_: The FC between left dorsolateral prefrontal cortex and right supramarginal gyrus; IA: ADHD-RS inattention factor.


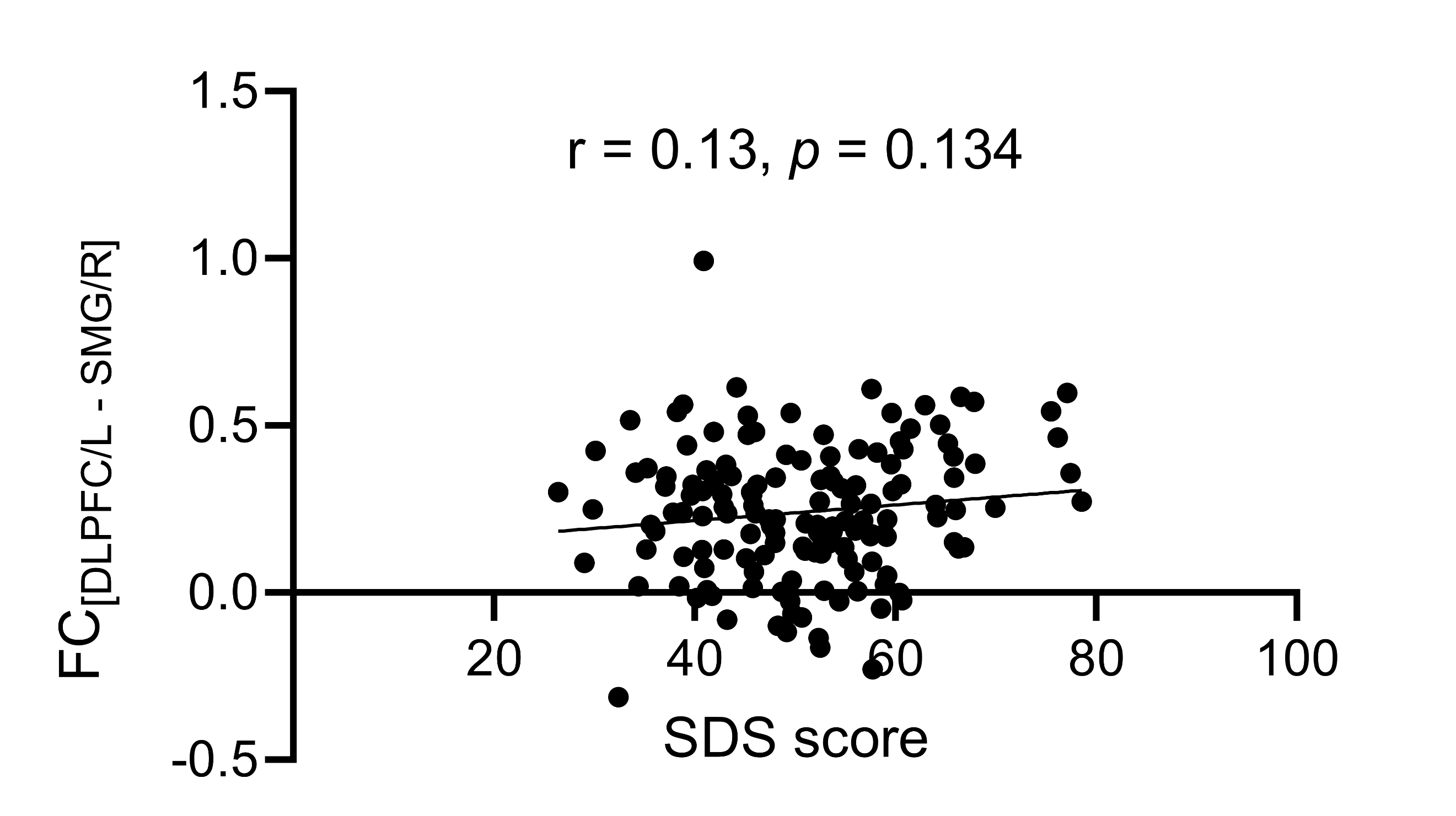


**Supplementary Fig. 2. The relationship between FC _[DLPFC/L - SMG/R]_ and SDS scores in ADHD patients.**

FC _[DLPFC/L - SMG/R]_: The FC between left dorsolateral prefrontal cortex and right supramarginal gyrus; SDS: Self-rating Depression Scale.


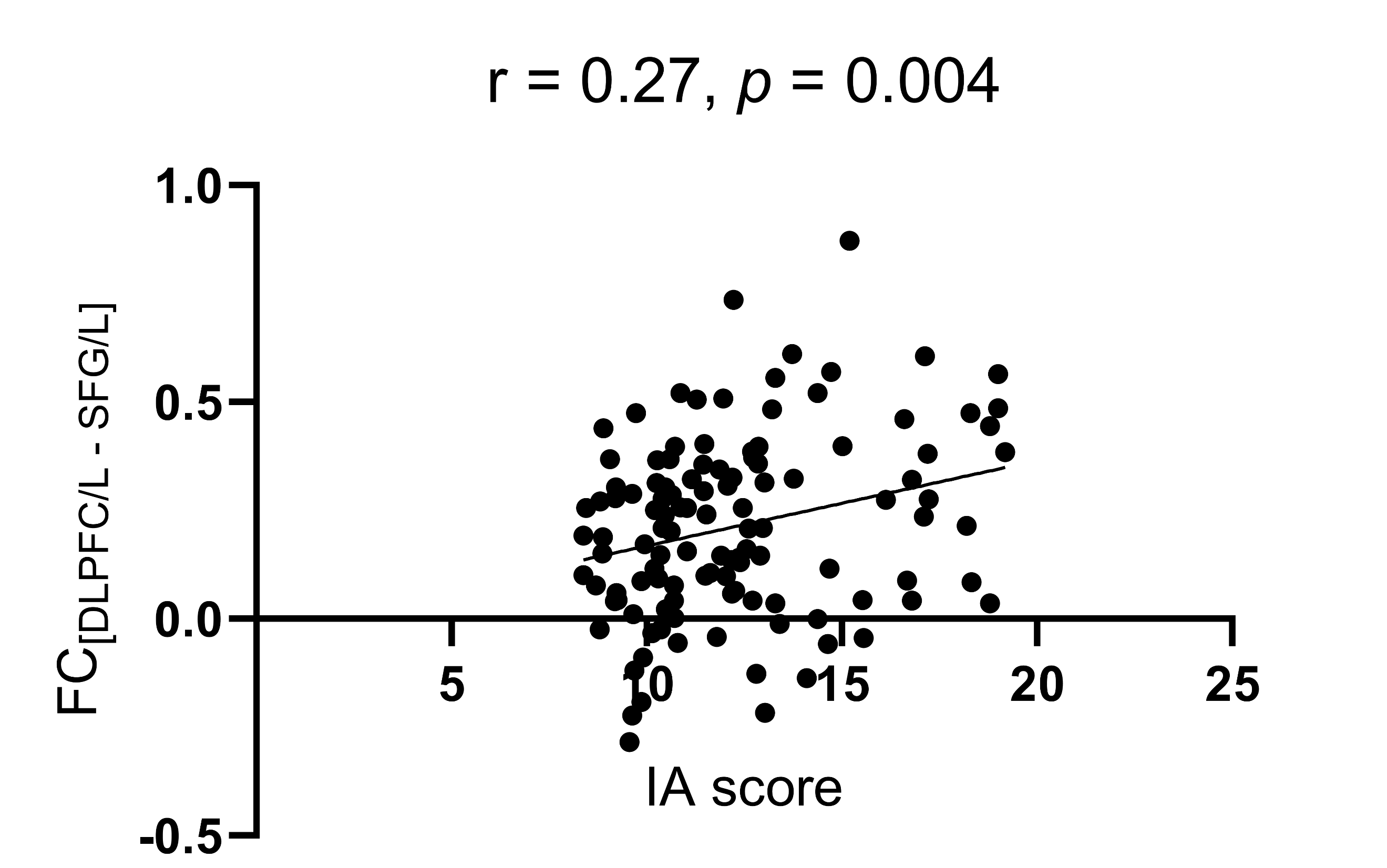


**Supplementary Fig. 3. The relationship between FC _[DLPFC/L - SFG/L]_ and IA scores in HC.**

FC _[DLPFC/L - SFG/L]_: The FC between left dorsolateral prefrontal cortex and left superior frontal gyrus; IA: ADHD-RS inattention factor.


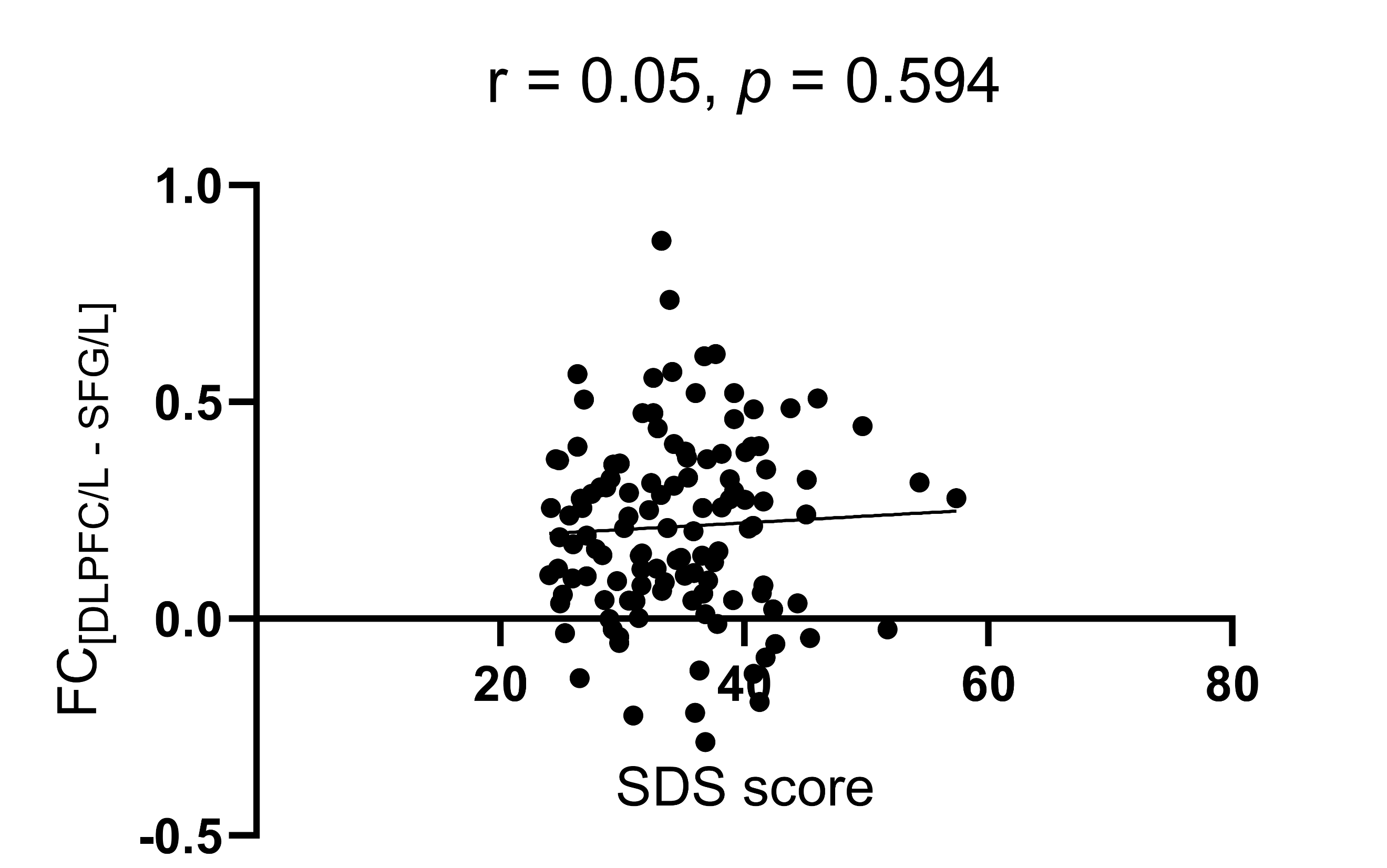


**Supplementary Fig. 4. The relationship between FC _[DLPFC/L - SFG/L]_ and SDS scores in HC.**

FC _[DLPFC/L - SFG/L]_: The FC between left dorsolateral prefrontal cortex and left superior frontal gyrus; SDS: Self-rating Depression Scale.

**Supplementary Table 2.** **Intergroup comparison in behavioral and imaging subgroup analysis**

|  |  | **ADHD+cMDD^1^** | **ADHD+pMDD^2^** | **ADHD-MDD^3^** | **HC^4^** | **F/χ2** | ***p*** | **Post hoc analyses** |
| --- | --- | --- | --- | --- | --- | --- | --- | --- |
| **Behavioral** **subgroup** | | | | | | | | |
|  | **n(Males, %)** | 49(24,49.0) | 76(26,34.2) | 145(74,51.0) | 139(55,39.6) | 7.47 | 0.058 | - |
|  | **Age(Mean ± SD)** | 26.24±5.99 | 25.97±5.94 | 26.45±5.57 | 25.36±3.22 | 1.17 | 0.323 | - |
|  | **IQ(Mean ± SD)** | 121.31±8.47 | 121.92±8.27 | 122.00±9.19 | 124.81±7.28 | 3.96 | 0.008 | 3＜4 |
|  | **IA(Mean ± SD)** | 19.31±3.76 | 18.97±4.31 | 18.60±4.18 | 12.35±3.07 | 84.00 | ＜0.001 | 1，2，3＞4 |
|  | **SDS(Mean ± SD)** | 65.54±9.73 | 54.29±11.19 | 49.39±10.70 | 35.13±7.43 | 143.61 | ＜0.001 | 1＞2＞3＞4 |
|  | **WM(Mean ± SD)** | 20.49±2.62 | 19.74±2.52 | 19.26±2.53 | 9.53±1.97 | 540.30 | ＜0.001 | 1，2，3＞4;1＞3 |
| **Imaging subgroup** | | | | | | | | |
|  | **n** | 23 | 40 | 83 | 120 |  |  |  |
|  | **FC _[DLPFC/L - SMG/R]_** | 0.26±0.21 | 0.26±0.25 | 0.19±0.18 | - | 2.82 | 0.063 | 1＞3; 2＞3 |

ADHD+cMDD^1^: ADHD patients currently comorbid with MDD; ADHD+pMDD^2^: ADHD patients previously comorbid with MDD; ADHD-MDD^3^: ADHD patients without MDD; HC^4^: healthy controls; IQ: Intelligence quotient; IA: Adult ADHD Rating Scale inattention factor; SDS: Self-rating Depression Scale; WM: Behavior Rating Inventory of Executive Function-Adult Version working memory factor.

**Supplementary Table 3. Comorbid status of adults with ADHD participants**

| **n** | **ADHD +MDD**  **whole group**  **(n = 125)** | **ADHD -MDD**  **whole group**  **(n = 145)** | **ADHD+MDD**  **Image subgroup**  **(n = 63)** | **ADHD-MDD**  **Image subgroup**  **(n = 83)** |
| --- | --- | --- | --- | --- |
| **All comorbidities (%)** | 48 (38.4) | 31(21.4) | 19(30.2) | 13(15.7) |
| **Persistent Depressive Disorder(%)** | 10(8) | 8(5.5) | 3(4.8) | 4(4.8) |
| **Panic Disorder(%)** | 5(4) | 2(1.4) | 3(4.8) | 2(2.4) |
| **Social Anxiety Disorder(%)** | 11(8.8) | 3(2.1) | 4(6.3) | 1(1.2) |
| **Specific Phobia(%)** | 6(4.8) | 6(4.1) | 2(3.2) | 5(6.0) |
| **Obsessive-Compulsive Disorder(%)** | 14(11.2) | 5(3.4) | 6(9.5) | 3(3.6) |
| **Generalized Anxiety Disorder(%)** | 14(11.2) | 7(4.8) | 5(7.9) | 2(2.4) |
| **Unspecified Anxiety Disorder(%)** | 5(4) | 6(4.1) | 2(3.2) | 1(1.2) |

**Sensitivity analyses with comorbid psychiatric status included as an additional covariate**

**Behavioral analyses**

The differences in WM impairment among the ADHD+MDD, ADHD-MDD, and HC groups were statistically significant (F = 708.90, *p* < 0.001). Specifically, both patient groups showed significantly more severe impairment than the HC group (all *p* < 0.001), and the ADHD+MDD group was significantly more impaired than the ADHD-MDD group (*p* = 0.019).

Subgroup analysis revealed significant differences in WM impairment among the ADHD+cMDD, ADHD+pMDD, ADHD-MDD, and HC groups (F = 475.61, *p* < 0.001). Specifically, all three patient groups exhibited significantly more severe impairment than the HC group (all *p* < 0.001). The ADHD+cMDD group was significantly more impaired than the ADHD-MDD group (*p* = 0.010), while no significant differences were found between the ADHD+cMDD and ADHD+pMDD groups (*p* = 0.579) or between the ADHD+pMDD and ADHD-MDD groups (*p* = 0.761).

Significant correlation was found between WM and SDS scores in ADHD patients, even when the IA score is also included as a covariant (r = 0.22, *p* < 0.001).

Hierarchical regression analyses in the ADHD group indicated that IA symptoms could explain 23.6% of the variance of WM (*p* < 0.001). With the existence of IA symptoms, the SDS score would explain 3.7% of the variance of WM independently (*p* < 0.001).

SDS score acted as a partial mediator between ADHD diagnosis and WM, even with IA symptoms controlled [β = -0.51(SE = 0.14), 95 % CI = (-0.79, -0.26)].

**Imaging analyses**

The FC _[DLPFC/L - SMG/R]_ was found to be higher in the ADHD+MDD group than in the ADHD-MDD group (*p* = 0.049). No difference was found (*F* = 2.04, *p* = 0.134) in FC _[DLPFC/L - SMG/R]_ among the ADHD+cMDD, ADHD+pMDD and ADHD-MDD groups.

The mediation analysis revealed that FC _[DLPFC(L)-SMG(R)]_ partially mediated the relationship between MDD comorbidity and WM impairment [β = -0.30, SE = 0.16, 95% CI = (-0.64, -0.00)]. However, this mediating effect became non-significant after additionally controlling for IA as a covariate [β = -0.21, SE = 0.13, 95% CI = (-0.47, 0.02)].

**Sensitivity analysis after excluding participants with other comorbid psychiatric conditions**

**Behavioral analyses**

Significant between-group differences in WM impairment were observed among the ADHD+MDD, ADHD-MDD, and HC groups (F = 805.07, *p* < 0.001). Post-hoc tests revealed that both patient groups showed significantly more severe WM impairment than the HC group (both *p* < 0.001), while no significant difference was found between the ADHD+MDD and ADHD-MDD groups (*p* = 0.823).

The subgroup analysis demonstrated significant WM impairment differences among ADHD+cMDD, ADHD+pMDD, ADHD-MDD, and HC groups (F = 536.00, *p* < 0.001). All three clinical subgroups exhibited significantly greater impairment than the HC group (all *p* < 0.001). However, no significant differences emerged in pairwise comparisons between any patient subgroups (all *p* = 1.000).

Significant correlation was found between WM and SDS scores in ADHD patients (r = 0.26, *p* < 0.001), even when the IA score is also included as a covariant (r = 0.23, *p* = 0.002).

Hierarchical regression analyses in the ADHD group indicated that IA symptoms could explain 22.0% of the variance of WM (*p* < 0.001). With the existence of IA symptoms, the SDS score would explain 4.0% of the variance of WM independently (*p* = 0.002).

SDS score acted as a partial mediator between ADHD diagnosis and WM [β = -0.96(SE = 0.23), 95 % CI = (-1.42, -0.51)], even with IA symptoms controlled [β = -0.50(SE = 0.16), 95 % CI = (-0.82, -0.21)].

**Imaging analyses**

No difference was found (*F* = 1.30, *p* = 0.257) in FC _[DLPFC/L - SMG/R]_ among the ADHD+MDD and ADHD-MDD groups.

After excluding all participants with other comorbid psychiatric conditions, the mediation analysis showed that FC [DLPFC(L)-SMG(R)] no longer significantly mediated the relationship between MDD comorbidity and WM impairment [β = -0.18, SE = 0.17, 95% CI = (-0.56, 0.16)].
